# The Role of Psychopathology and Emotion Regulation in the Intergenerational Transmission of Childhood Abuse: A Family Study

**DOI:** 10.1177/10775595231223657

**Published:** 2024-02-01

**Authors:** Cosima A. Nimphy, Marie-Louise J. Kullberg, Katharina Pittner, Renate Buisman, Lisa van den Berg, Lenneke Alink, Marian Bakermans-Kranenburg, Bernet M. Elzinga, Marieke Tollenaar

**Affiliations:** 1 Department of Clinical Psychology, Institute of Psychology, 4496 Leiden University, Leiden, The Netherlands; 2 Institute of Medical Psychology, 14903 Charité–Universitätsmedizin Berlin, Corporate member of Freie Universität Berlin and Humboldt-Universität Berlin, Leiden, The Netherlands; 3 Institute of Education and Child Studies, 4496 Leiden University, Leiden, The Netherlands; 4 Leiden Institute for Brain and Cognition (LIBC), Leiden, The Netherlands; 5 Yulius, Rotterdam, The Netherlands; 6 William James Center for Research, ISPA –University Institute of Psychological, Social and Life Sciences, Lisbon, Portugal; 7 Department of Psychology, Personality, Social and Developmental Psychology, Stockholm University, Stockholm, Sweden

**Keywords:** intergenerational transmission, abuse, psychopathology, emotion regulation, externalizing problems

## Abstract

Previous studies have shown that parents with a history of childhood abuse are at increased risk of perpetrating child abuse. To break the cycle of childhood abuse we need to better understand the mechanisms that play a role. In a cross-sectional extended family design including three generations (*N* = 250, 59% female), we examined the possible mediating role of parental psychopathology and emotion regulation in the association between a history of childhood abuse and perpetrating child abuse. Parents’ own history of childhood abuse was associated with perpetrating abuse toward their children, and externalizing (but not internalizing) problems partially mediated this association statistically. Implicit and explicit emotion regulation were not associated with experienced or perpetrated abuse. Findings did not differ across fathers and mothers. Findings underline the importance of (early) treatment of externalizing problems in parents with a history of childhood abuse, to possibly prevent the transmission of child abuse.

Childhood maltreatment, including both abuse and neglect, is a problem with worldwide prevalence. The self-reported rate of abuse is between 23% and 36% and the self-reported rate of neglect ranges between 16% and 18% (Stoltenborgh et al., 2015). Empirical evidence suggests that experiences of childhood maltreatment can lead to pervasive mental health problems, including internalizing and externalizing problems, as well as substance abuse (Hughes et al., 2017; Norman et al., 2012). These mental health problems tend to persist into adulthood (Heim et al., 2010). Furthermore, research suggests that a history of childhood maltreatment in parents may lead to a higher risk for parents to perpetrate maltreatment towards their children (Assink et al., 2018; Madigan et al., 2019; Stith et al., 2009), also coined as intergenerational transmission of child maltreatment (ITCM) (Yang et al., 2018).

Recent meta-analyses do indicate the above-chance presence of intergenerational transmission of childhood maltreatment (Assink et al., 2018; Madigan et al., 2019). Furthermore, Van IJzendoorn and colleagues (2020) synthesized the findings of meta-analyses that investigated risk factors or effects of interventions on the perpetration of childhood maltreatment and found that parents’ experienced childhood maltreatment (such as abuse) is a robust predictor of later perpetration of childhood maltreatment. In the sample that is part of the current study (Buisman et al., 2020; Pittner et al., 2020; Van den Berg et al., 2018, 2019), parents who had been (physically or emotionally) abused in childhood were also more likely to abuse their children. This association was found when incorporating both self-report as well as other-reports from parents and children (i.e., with a multi-informant measure), whereas intergenerational transmission of neglect was only found when using self-report and not multi-informant measurements (Buisman et al., 2020). Given the more robust evidence for intergenerational transmission of abuse rather than neglect in our sample, this study focused on the transmission of abusive behavior. To break the cycle of childhood abuse, we need to examine the mechanisms that may play a role.

Psychopathology, including both internalizing and externalizing disorders, is one of the most frequently studied mechanisms in intergenerational transmission of abuse (Greene et al., 2020). It has been suggested that psychosocial stress in childhood, such as having a history of child abuse, may induce chronic and dysfunctional over- and underactivity of the psychobiological systems that are responsible for regulating stress responses (Alink et al., 2019). This may contribute to heightened internalizing/externalizing problems. In turn, parents with internalizing/externalizing problems might have decreased capacities to provide supportive and nurturing responses to their children, to regulate parenting-related stress, and as a result might be at increased risk of perpetrating abuse (Lamela & Figueiredo, 2013). Despite consistent findings that psychopathology is linked to both experienced and perpetrating childhood abuse (Scott et al., 2010; Stith et al., 2009), findings on the role of psychopathology as a mediator of intergenerational transmission of abuse have been mixed (Greene et al., 2020). While some studies suggest that parents’ psychopathology may be involved in the intergenerational transmission of abuse (Lamela & Figueiredo, 2013; Milner et al., 2010; Yang et al., 2018), others did not find support for this hypothesis (Banyard et al., 2003). A reason for these mixed findings may be that psychopathology has been operationalized differently across the aforementioned studies, such as general mental health difficulties, specific disorders (e.g., depression or substance abuse), or internalizing and externalizing problems. There is some evidence that especially internalizing problems (e.g., psychosomatic or post-traumatic stress [PTSD] symptoms) or internalizing disorders (such as major depressive disorder) increase the risk of intergenerational transmission of abuse (Lamela & Figueiredo, 2013; Milner et al., 2010; Yang et al., 2018).

While there is substantial research on the role of internalizing problems, studies investigating the role of externalizing psychopathology in the intergenerational transmission of abuse are scarce. One longitudinal study (Augustyn et al., 2019) found that delinquency, which can be seen as externalizing symptomatology, mediated intergenerational transmission of maltreatment. However, the study oversampled male participants (3:1 ratio) and it could not be tested whether the findings varied depending on sex. By investigating the role of parental sex in intergenerational transmission of abuse in our study, we aim to gain more insight into the generalizability of previous findings. Moreover, given the high correlation between internalizing and externalizing problems in previous adult samples (correlation of .74 in a study of more than 10,000 adults from 17 different societies, Rescorla et al., 2016), studies investigating the role of internalizing or externalizing problems separately are unable to disentangle their unique contribution in their association with perpetrating child abuse. By including both internalizing and externalizing problems as parallel mediators in one statistical model, we can get more insight into which of these factors drives the association to perpetrated child abuse.

Another explanation for the discrepant findings regarding the role of psychopathology in intergenerational transmission of abuse might be that a transdiagnostic factor, that has been linked to both internalizing and externalizing problems, underlies this association. Impaired emotion regulation is a plausible candidate for such an underlying factor. Emotion regulation refers to “the processes by which individuals influence which emotions they have, when they have them, and how they experience and express these emotions” (Gross, 1998). Impaired emotion regulation abilities are a risk factor for various psychopathologies such as anxiety disorders (Suveg et al., 2005), depression (Campbell-Sill & Barlow, 2007), and borderline personality disorder (Gratz et al., 2006), and have been found to explain part of the association between childhood abuse and internalizing disorders (Milojevich et al., 2019). Children who grow up in an abusive environment are more alert and vigilant to their parent’s actions to assess the risk of being abused and can exhibit increased levels of attention to threat (McCrory et al., 2017). This increased threat processing may decrease the availability of psychological resources for other social information processing. Some children may have fewer resources to divert attention from negative stimuli and thereby have problems in regulating arousal or impulsive (re)actions to negative stimuli (Cupach & Olson, 2006). Additionally, abused children may also learn maladaptive emotion regulation strategies via social learning processes from their parents (Bandura & Walters, 1977; Eisenberg et al., 2001). Recent studies found that experienced childhood maltreatment (including both abuse and neglect) is linked to emotion dysregulation (Warmingham et al., 2020) and more frequent use of maladaptive emotion regulation strategies such as suppressing emotions and rumination (Weissman et al., 2019). At the same time, problems in emotion regulation can also impact parenting and may increase the risk of abuse. If a parent is unable to regulate their emotional responses to stress, they are more likely to divert to harsh and abusive reactions towards their children than a parent with better emotion regulation abilities (Lowell & Renk, 2017; Rodriguez et al., 2017).

Studies indeed suggest a link between experienced abuse and emotion dysregulation (for meta-analysis see Lavi et al., 2019), as well as between emotion dysregulation and perpetrating abuse (risk) (for meta-analysis see Lavi et al., 2021). To our knowledge, only one study has so far reported that the link between maternal history of childhood abuse and the perpetration of child abuse is mediated by emotional dysregulation (Smith et al., 2014). However, this study only investigated the mediating role of emotion regulation in the intergenerational transmission of abuse in mothers. Therefore, it is unclear to what extent this finding generalizes to the intergenerational transmission of abuse in fathers. Hence, in the current study, we will investigate the role of emotion regulation in the intergenerational transmission of abuse in both mothers and fathers and assess whether the findings vary depending on parental sex.

Given the vast breadth of definitions and operationalizations of emotion regulation, it is still unclear which constructs of emotion regulation may specifically be related to intergenerational transmission of abuse. Emotion regulation can be categorized into explicit emotion regulation and implicit emotion regulation (Etkin et al., 2015; Gyurak et al., 2011). Explicit emotion regulation can be defined as consciously changing the meaning of a stimulus, which evokes the emotion. It requires a certain extent of emotional awareness, as well as emotion monitoring. Usually, explicit emotion regulation strategies are measured by administering self-reports, as they assess the subjective insights on individuals’ cognitive emotion regulation strategies. In contrast, implicit emotion regulation includes automatic processes that regulate emotions without conscious effort from the individual. Implicit emotion regulation does not require emotional awareness and emotion monitoring, and thus cannot be measured by self-report. In our study, we operationalized implicit emotion regulation as an individual’s ability to suppress threat information during a working memory performance task. Previous literature investigating the association between childhood abuse and emotion regulation assessed emotion regulation mainly with self-report questionnaires, i.e., explicit emotion regulation (Garnefski et al., 2017; Garnefski & Kraaij, 2007; Gratz & Roemer, 2004; Shields & Cicchetti, 1997). Also, the above-mentioned study by Smith et al. (2014) that found emotion regulation mediating intergenerational transmission of abuse, only assessed explicit emotion regulation with a questionnaire. No study has yet replicated the study by Smith et al. (2014) or investigated the role of implicit emotion regulation in intergenerational transmission of abuse. As mentioned in Lavi et al.’s recent meta-analysis (2021), more studies investigating the relation between parents’ emotion regulation and their perpetration of childhood maltreatment are needed that differentiate between different modes of emotion regulation and focus on specific types of maltreatment (i.e., abuse) rather than mixed subtypes (i.e., abuse and neglect combined).

## Aim and Hypotheses

This study aims to examine the mediating role of psychopathology and emotion regulation in the association between parental experienced and perpetrated child abuse. We will specifically differentiate between internalizing and externalizing psychopathology and implicit and explicit emotion regulation. As our constructs were assessed cross-sectionally (emotion regulation and psychopathology) and retrospectively (experienced and perpetrated abuse) on the same assessment day, we can only assess and interpret statistical mediation. We hypothesize that both internalizing and externalizing psychopathology and implicit and explicit emotion dysregulation mediate intergenerational transmission of abuse. Additionally, we will explore whether these mechanisms are similar for mothers and fathers.

## Method

### Participants

Participants of this study were part of the Three Generation Parenting (3G) study, which investigated the relations between genetic, environmental, and methodological factors in the transmission of parenting styles, emotional regulation, and stress across generations (Buisman et al., 2020). Participants of the 3G study (*N* = 395) were recruited via three participant pools: Firstly, the Netherlands Study of Depression and Anxiety (NESDA; Penninx et al., 2008); secondly, the Longitudinal Internet Studies for the Social Sciences (LISS panel; Scherpenzeel & Toepoel, 2012), and lastly a study on parenting (Joosen et al., 2013). If a participant agreed to take part in the study and gave permission, family members of this participant and their partner were also asked to participate. These included parents, children, siblings (and their partners), nephews, and nieces. To be included in the 3G study, participants had to be at least 7.5 years old, and within each family, at least two first-degree relatives from two generations had to agree to participate. For this specific study, we only included participants from the larger 3G sample if they were older than 18 years, had children of their own, and reported on both experienced and perpetrated abuse (*n* = 250). Sample characteristics of our study can be found in Table 1. Our sample consists of 147 women (59%) and 103 men. On average, participants were 51 years old. The majority (more than 50%) of participants in the first and second generations had two children. Ninety-six percent of participants are Caucasian, two percent from the Netherlands Antilles/Surinam, and two percent from another ethnicity.

**Table 1.** Sample Characteristics.


	M (SD)/% (*n*)	*N*
Age *M(SD)*	51.3 (13.7)	250
Female % (*N*)	58.8 (147)	
Highest education		
Lower vocational education	71.6 (*179*)	
Intermediate vocational education	19.6 (*49*)	
Higher vocational or scientific education (university)	6.0 (*15*)	
Brutto yearly income of household		
Less than €15.000	3.2 (*8*)	
€15.000 - €24.999	14.4 (*36*)	
€25.000 - €34.999	16.8 (*42*)	
€35.000 - €44.999	14 (*35*)	
€45.000 - €54.999	12 (*30*)	
€55.000 - €64.999	7.2 (*18*)	
€65.000 of meer	12.8 (*32*)	
Missing	16.8 (*42*)	
Experienced child abuse *M (SD)*	1.6 (.52)	250
Perpetrated child abuse *M (SD)*	1.5 (.29)	250
ASR internalizing problems *M (SD)*	53.3 (10.0)	239
ASR externalizing problems *M (SD)*	43.8 (5.8)	239
EWMT emotion regulation *M (SD)*	.03 (.92)	119
CERQ *M (SD)*	25.9 (7.2)	238

*Notes*: *M* = Mean, *SD* = Standard deviation, ASR = Adult Self Report, CERQ = Cognitive Emotion Regulation Questionnaire, EWMT = Emotional Working Memory Task.

### Procedure

Participants were invited to the lab for one or two days, depending on family size and composition. During the visits, participants individually filled in questionnaires, performed computer tasks, and completed several interaction tasks together with their family members. The study was approved by the ethics committee of the Leiden University Medical Center (reference number: P11.134)*.* Informed consent was obtained from all participants before participation. As compensation for participation, adult participants received 50Euros for one lab visit and up to 100Euros for both lab visits together. Children received age-adequate compensation and all traveling expenses were paid.

### Materials

The current paper investigated the association between childhood abuse, psychopathology, and emotion regulation across three generations of the 3G study. Therefore, the methods described are focussed on the relevant materials. Information on all variables of the 3G study can be found in previously published papers (see Buisman et al., 2019, 2020; Pittner et al., 2020).

#### Child Abuse

To assess experienced as well as perpetrated child abuse, the Parent-Child Conflict Tactics Scales (CTS-PC; Straus et al., 1998) were administered. To assess the amount of (perpetrated or experienced) abuse, we first averaged the item scores of the subscale Psychological Aggression (5 items) and the subscale Physical Assault (13 items), and then computed an average score. All items were scored on a 5-point Likert scale from 1 = never to 5 = almost always. Participants reported the extent to which they experienced abuse inflicted by their father and/or mother (depending on who they were raised by) before the age of 18 years. Participants with children were also assessed on the extent they perpetrated abuse towards (each of) their child(ren) before the age of 18. In our study, based on the experienced abuse items, for at least one of the items of each scale, self-reported experienced abuse occurred in 90% more than once, 6% once, and only 4% never (see Table S1 in the supplementary material for more information on self-reported occurrence rates of the emotional and physical abuse subscales). The agreement on abuse scores between father-child (ICC = .32), mother-child (ICC = .28), and father-mother (ICC = .31) in the 3G study was moderate, which shows inter-reporter differences as well as overlap (see Buisman et al., 2020). To calculate the final experienced and perpetrated abuse scores in this study, we used the abuse information from multiple informants where available. That is, perpetration as reported by the parent’s father and mother averaged with the parent’s self-report for experienced abuse, and self-report by the parent combined with reports by their child(ren) for perpetrated abuse. Per subscale, if both parents reported perpetrating child abuse, the higher score of mother or father was used, and if multiple children reported on one parent, the higher score was used. If information from other reporters was not available, only self-report was used (for calculation of final scores see Buisman et al., 2019, 2020; Pittner et al., 2020)*.* In total, 31% of experienced abuse scores were based on combined scores and 69% on self-report scores, whereas 86% of the perpetrated abuse scores were based on combined scores and 14% on self-report. Higher final experienced and perpetrated child abuse scores indicate more frequent experiences and or perpetration of child(hood) abuse. Descriptive information on the (combined) abuse scores for separate subscales can be found in Table 2. In the 3G study, the internal consistencies of the experienced abuse subscales were .92 for mother physical abuse, .92 for father physical abuse, .78 for mother emotional abuse, and .73 for father emotional abuse. The internal consistencies of the perpetrated abuse subscales were .71 for the physical abuse scores of the first child, .76 for the second child, .69 for the emotional abuse scores of the first child, and .66 for the second child (see Buisman et al., 2020).

**Table 2.** Descriptive Information on Abuse Scores.

	*N*	Minimum	Maximum	*M*	*SD*
Perpetrated emotional abuse	250	1	3.4	1.74	.46
Perpetrated physical abuse	250	1	2.1	1.2	.19
Experienced emotional abuse	250	1	4.2	1.9	.67
Experienced physical abuse	250	1	5	1.39	.45

*Notes*: *M* = Mean, *SD* = Standard deviation.

#### Psychopathology

To assess symptoms of psychopathology, the Adult Self Report (ASR; Achenbach et al., 2003) was administered. Items are scored on a scale ranging from not true (0) to often true (2). To assess internalizing psychopathology, we calculated a total score by adding item scores of the following ASR subscales: (1) anxious and/or depressed, (2) withdrawn, and (3) somatic complaints. To assess externalizing psychopathology, we calculated a total of the following ASR subscales: (4) aggressive behavior, (5) rule-breaking, and (6) intrusive behavior. Higher scores on the internalizing and externalizing subscales indicate more internalizing and externalizing symptoms. The internalizing subscale consists of 39 items and has a reliability of .91 in the current sample, while the externalizing scale entails 35 items and has a reliability of .81.

#### Emotional Regulation

To assess implicit and explicit emotion regulation, two measures were administered, namely an Emotional Working Memory Task and the Cognitive Emotion Regulation Questionnaire.

#### Implicit Emotion Regulation

Implicit emotion regulation was assessed with the Emotional Working Memory Task (EWMT; see Oei et al., 2009; Krause-Utz et al., 2012; Krause-Utz et al., 2014). This task measures the extent of distraction by negative emotional information during a working memory performance task. The current version of the EMWT task consisted of 96 trials and lasted approximately 10 minutes. At the beginning of each trial, a black fixation cross was displayed for 750 ms, after which 1, 2, 3, or 4 target letters were presented below each other for 1000 ms, which should be memorized. There was a delay interval of 1500 ms between the display of the target list and the recognition list. In half of the trials, an emotional distractor in the form of a negative picture was shown during the delay interval, while in the other half a neutral picture was displayed. Pictures were chosen from the International Affective Picture System (IAPS, Lang et al., 2008). Next, the presence or absence of one of the target letters in a recognition list of one to four letters should be indicated by pressing a yes or no button within a 2000 ms period. In 50% of the trials, a target letter was present, and in the other 50% letters were absent. The maximum comparison load was 8 (e.g., remembering two letters and recognizing them among four letters). During the task, participants were instructed to stay focused on the fixation cross in the center of the screen, ignore the pictures, and focus on the working memory task.

Performance on this task was measured by computing the participant’s response times (RT) of correct trials (see Krause-Utz et al., 2012; Krause-Utz et al., 2014, on which this method was based). To measure distraction during the task by the emotionally negative pictures for each individual, we wanted to assess the length of the RTs of the negative (Mean (*SD*) RT = 1304.11 (273.85)) versus the neutral picture trials (Mean (*SD*) RT = 1251.42 (278.85)) for the correct trials, while correcting for a person’s general RT. To this end, we performed a regression analysis in which we predicted the RT of the negative picture trials from the RT of the neutral picture trials and calculated the standardized residuals from this regression analysis. These residuals indicate the standardized difference between the RT of task performance after seeing negative versus neutral pictures. A larger residual score indicates that a participant was more distracted by the negative stimulus than would be predicted by the regression model. This larger residual score indicates increased interference by negative emotional stimuli, which can be interpreted as maladaptive emotion regulation due to increased threat processing.

#### Explicit Emotion Regulation

Explicit Emotion regulation was assessed with the Cognitive Emotion Regulation Questionnaire (CERQ; Garnefski & Kraaij, 2007). The CERQ is a self-report questionnaire, which measures the frequency of nine cognitive emotion regulation strategies. This questionnaire is scored on a 5-point Likert Scale from (almost) never (1) to (almost) always (5). As the subscales rumination, catastrophizing, and self-blame are most strongly related to psychopathology and are highly correlated (Garnefski et al., 2017), we combined these three subscales into one maladaptive emotion regulation scale. The higher the total score, the more frequently participants use maladaptive emotion regulation strategies. The internal consistency of the current maladaptive emotional dysregulation scale was good, with a Cronbach’s alpha of .84.

#### Covariates

Demographic information, such as age, sex, and household social economic status (SES), was acquired with questionnaires. SES was assessed with questions on education level, income, and occupation. The scores on these questions were standardized and then summed. A SES score higher than zero indicates that the participant’s SES score is higher than the average SES in the sample (see Buisman et al., 2020).

### Data Analyses

First, the data were explored to check means, standard deviations, outliers, and whether the variables were normally distributed. Then, we examined the Pearson correlations between experienced abuse, perpetrated abuse, emotion regulation (implicit and explicit), and psychopathology (internalizing and externalizing) and the covariates. Next, a structural equation model was computed in R, using the lavaan-package (.6–3 (Rosseel, 2012) in R version 3.5.1 (R Core Team, 2018) to simultaneously test the associations between experienced abuse, internalizing/externalizing psychopathology, and perpetrated abuse. Additionally, a second structural model was run to test the associations between experienced abuse, implicit/explicit emotion regulation, and perpetrated abuse. For both models, we included the significant covariates (based on Pearson correlations with the variables of interest) in the final analyses to control for their effects. Due to the significant association between internalizing and externalizing psychopathology, we included their covariation in the first model. Since implicit and explicit emotion regulation were not significantly associated with each other, we did not add their covariation to the second model. We chose to test two models (one for emotion regulation and one for psychopathology), as one of the prerequisites of parallel mediation is the assumption that the mediators are not causally influencing each other (Hayes, 2013). However, emotion dysregulation has been strongly linked to and mentioned as a potential cause of development of the psychopathology (Aldao et al., 2010), and hence we studied them in separate models. Furthermore, we controlled for the nestedness of the data (due to the family structure) by calculating robust standard errors using the R package lavaan.survey. This entails the aggregation of the structural model parameter estimates over clusters. For more information on the lavaan.survey package and its use, see Muthen and Satorrah (1995) and Oberski (2014). The model parameters were calculated with a maximum likelihood estimator (Rosseel, 2012). Of the 250 participants, we miss 4.4% of the Adult Self Report (internalizing and externalizing problems) scores (11/250). Additionally, we miss 4.8% of the Cognitive Emotion Regulation Questionnaire scores (explicit emotion regulation) (12/250). and 52.4% on the EWMT (implicit emotion regulation (119/250). Full Information Maximum Likelihood (FIML) was used to handle missing values. To assess model fit, we inspected the Chi-Squared (χ2) value, the comparative fit index (CFI), and the root mean square error of approximation (RMSEA). According to Hu and Bentler (1999), a CFI above .95, a RMSEA below .05, and a non-significant value of χ2 statistic are considered to be a good fit between the model and the observed data. To explore whether these mechanisms are similar for mothers and fathers, we ran a multiple-group model with sex as a group variable and compared the constrained model, fixing regressions to be equal across groups, to the unconstrained model with a chi-square difference test.

## Results

Pearson’s correlations (see Table 3) revealed a significant association between experienced and perpetrated abuse, between internalizing and externalizing problems, which were both associated with experienced and perpetrated abuse, as well as between maladaptive emotion regulation strategies and internalizing/externalizing problems. Interestingly, implicit and explicit emotion regulation were not significantly associated with each other. Due to significant associations between age, SES, and sex and variables of interest, we included age and SES as covariates in model 1 and age and sex in model 2.

**Table 3.** Pearson Correlations Between Study Variables.

	1	2	3	4	5	6	7	8	9
1. Age	1								
2. Sex (male)	−.03	1							
3. Household SES	−.26^**^	−.10	1						
4. Experienced abuse	.10	.03	−.05	1					
5. Perpetrated abuse	.20^**^	.06	−.11	.41^**^	1				
6. IP (ASR)	.07	.13	−.14^*^	.31^**^	.20^**^	1			
7. EP (ASR)	−.06	−.10	−.04	.29^**^	.28^**^	.57^**^	1		
8. EER (CERQ)	−.03	.08	−.05	.09	.06	.49^**^	.32^**^	1	
9. IER (EWMT)	.11	−.23^*^	.06	.10	.01	−.05	.04	−.12	1

*Notes*: ** = Correlation is significant at the .01 level (2-tailed), * = Correlation is significant at the .05 level (2-tailed). IP = internalizing problems, EP = externalizing problems, CERQ = Cognitive Emotion Regulation Questionnaire, EWMT = Emotional Working Memory Task.

**Figure 1.**
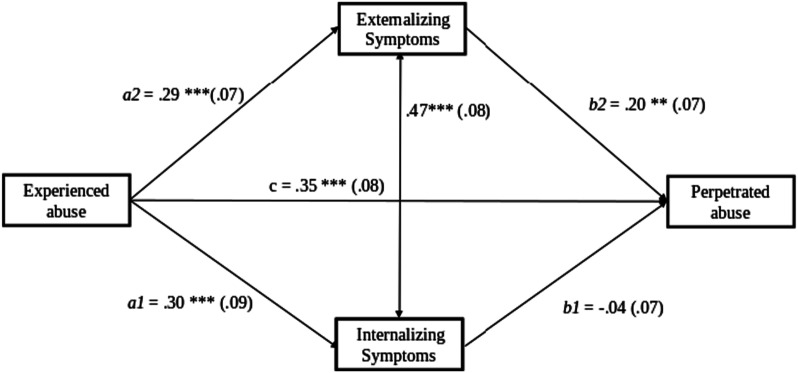
Model 1: Internalizing and externalizing problems as mediators in the association between the history of childhood abuse and perpetrating child abuse. Note: adjusted standard errors in parentheses, model is controlled for household SES and age.

In the first structural equation model, the associations between experienced abuse, perpetrated abuse, and externalizing and internalizing problems were simultaneously tested (see Table 4, Figure 1). Internalizing and externalizing problems were significantly correlated, *β* = .47, *SE* = .08, *CI* [.31, .64], *p* < .001, and experienced abuse was significantly associated with perpetrating abuse, *β* = .35, *SE* = .08, *CI* [.19, .51], *p* < .001. Moreover, parents who experienced more abuse in their childhood reported more externalizing (*β* = .29, *SE* = .07, *CI* [.15, .42], *p* < .001) and internalizing problems (*β* = .30, *SE* = .09, *CI* [.13, .46], *p* = .001). However, while externalizing problems were positively related to perpetrating abuse (*β* = .20, *SE* = .07, *CI* [.07, .34], *p* = .003), internalizing problems were not (*β* = −.04, *SE* = .07, *CI* [-.18, .11], *p* = .580). The indirect effect via externalizing problems in the association between experiencing abuse in childhood and being abusive towards the children was significant, *β* = .06, *SE* = .03, *CI* [.01, .11], *p* = .022. The indirect effect via internalizing problems in the association between having experienced abuse in childhood and being abusive towards your children was not significant, *β* = −.01, *SE* = .02, *CI* [-.06, .03], *p* = .622. In the model, we controlled for the effects of age and SES. The model had an acceptable fit, indicating it described the data adequately (χ2 = 4.83, *df* = 4, *p* = .305, CFI = .995, RMSEA = .029).

**Table 4.** Internalizing and Externalizing Problems as Mediators in the Association Between the History of Childhood Abuse and Perpetrating child Abuse.


path	Beta	SE	adj SE	*p*
Total effect	.40	.07		<.001
Direct effect: EA-- > PA	.35	.08	.08	<.001
a1 (EA-- > IP)	.30	.09	.09	<.001
a2 (EA-- > EP)	.29	.07	.07	<.001
b1 (IP-- > PA)	−.04	.07	.07	.580
b2 (EP-- > PA)	.20	.07	.07	.003

Notes. adj *SE* = standard error adjusted for dependency in the data within families, *SE* = standard error, EA = experienced abuse, PA = perpetrated abuse, IP = internalizing problems, EP = externalizing problems, model is controlled for SES and age.

**Figure 2.**
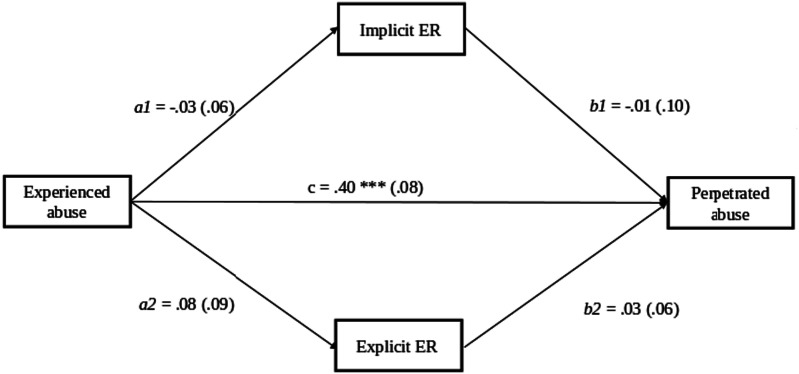
Model 2: Implicit and explicit emotion regulation as mediators in the association between the history of childhood abuse and perpetrating child abuse. *Note*: adjusted standard errors in parentheses, model is controlled for sex and age.

In the second structural equation model, the associations between experienced abuse, perpetrated abuse, and explicit and implicit emotion regulation were tested simultaneously (see Table 5, Figure 2). Experienced abuse was again significantly associated with perpetrating abuse, *β* = .40, *SE* = .08, *CI* [.25, .54], *p* < .001, but there was no association between implicit and explicit ER. In contrast to our hypotheses, we did not find an association between experienced childhood abuse and explicit emotion regulation strategies (*β* = .08, *SE* = .09, *CI* [-.08, .25], *p* = .370) nor between experienced childhood abuse and implicit emotion regulation (*β* = −.03, *SE* = .06 *CI* [-.15, .09], *p* = .589). Furthermore, neither explicit (*β* = .03, *SE* = .06, *CI* [-.10, .17], *p* = .618) nor implicit emotion regulation was associated with perpetrating abuse (*β* = −.01, *SE* = .01, *CI* [-.27, .25], *p* = .920). Hence, no indirect effect of implicit and explicit emotion regulation in the association between having experienced abuse in childhood and being abusive towards their children was found, both *ps* > .647. In the model, we controlled for the effects of age and parent sex. The model had an acceptable model fit, *χ2* = 9.72, *df* = 5, *p* = .0843, CFI = .90, RMSEA = .061.

**Table 5.** Implicit and explicit emotion Regulation as Mediators in the Association Between the History of Childhood Abuse and Perpetrating child Abuse.


path	Beta	SE	adj SE	*p*
Total effect	.41	.07		<.001
Direct effect: EA-- > PA	.40	.08	.08	<.001
a1 (EA-- > IER)	−.03	.06	.06	.579
a2 (EA-- > EER)	.08	.08	.09	.370
b1 (IER-- > PA)	.01	.13	.10	.920
b2 (EER-- > PA)	.03	.07	.06	.618

Notes. adj *SE* = standard error adjusted for dependency in the data within families, *SE* = standard error, EA = experienced abuse, PA = perpetrated abuse, IER = implicit emotion regulation, EER = explicit emotion regulation, model is controlled for sex and age.

To test for differences between fathers and mothers, we ran a multiple-group model with sex as the grouping variable. For both models, the chi-square difference test indicated that the constrained model, fixing regressions to be equal across groups, did not differ from the unconstrained model 1 (Δ *χ2* = 3.67, *p* = .452). This suggests that the associations between experienced abuse, perpetrated abuse and psychopathology did not differ across sex. The same was the case for model 2, Δ *χ2* = 2.68, *p* = .612.

As a sensitivity analysis, we ran a mediation model including all four mediators. Results are similar and have the same conclusions as those from the separate psychopathology and emotion regulation models. However, the model does not have an adequate fit (*χ2 =* 30.77, *df* = 15, *p* = .009, CFI = .934, RMSEA = .065) (see Figure S1 in Supplementary Material).

## Discussion

The present study investigated the role of internalizing and externalizing problems as well as implicit and explicit emotion regulation in the intergenerational transmission of childhood abuse in an extended family design including three generations. Findings show that a parental history of childhood abuse is associated with perpetrating abuse and that externalizing problems of the parent partially explained this association. While internalizing problems were associated with experienced abuse, they were not associated with perpetrating abuse. Hence, intergenerational transmission of abuse might not be explained by internalizing problems but might be attributed to heightened levels of externalizing problems. Additionally, emotion dysregulation was neither associated with experienced nor perpetrated abuse in the current sample.

In line with previous findings, our study found that externalizing symptomatology was related to both having experienced childhood abuse and perpetrating abuse towards their children. Furthermore, we also found that the association between having experienced and perpetrating childhood abuse was partially explained by externalizing problems, which included aggressive behavior, rule-breaking, and intrusive behavior (Augustyn et al., 2019; Stith et al., 2009). As discussed by Augustyn et al. (2019), modeling of parent's abusive behaviors might influence children’s behavior. Over time children might see these abusive and aggressive behaviors as an appropriate response to difficulties (Capaldi et al., 2009; Pears & Capaldi, 2001). Therefore, individuals who learn to turn outwards with aggression to difficulties, rather than react with for example withdrawal, might also be more likely to direct these behaviors toward their children, which might continue the cycle of abuse. These patterns are in line with previous findings from the current study sample showing that the father’s externalizing problems were associated with more observed negativity toward offspring and that the offspring’s externalizing problems were related to more observed negativity toward their father during a behavioral conflict interaction task (Kullberg et al., 2023). Nevertheless, given the retrospective and cross-sectional design of our study, we have to be careful in interpreting directionality and causality. That is, externalizing problems might not be a mediator but related to another (third) variable, such as an underlying genetic family trait, that may explain the association between experienced and perpetrating abuse. As such, child behavioral difficulties may elicit more harsh parenting. Furthermore, from a stress-exacerbation standpoint (Tucker et al., 2014), the parent-child relationship, family dysfunction, or parent mental health problems could exacerbate the negative effects of parents’ own experienced childhood abuse on parents’ use of abusive/harsh parenting strategies. Hence, we can only speculate on the direction of the effects and future studies should try to test the directionality of the interactions between psychopathology and parent-child interactions.

Moreover, our findings showed no differences between mothers and fathers across the investigated associations in our mediation model assessing whether externalizing problems explain significant variance in the association between childhood experiences of abuse and perpetrating child abuse. This extends the findings of Augustyn et al. (2019), by suggesting that externalizing symptomatology might mediate intergenerational transmission of abuse for both mothers and fathers similarly.

As previous studies suggested that internalizing symptoms mediate the link between having experienced and (risk of) perpetrating childhood abuse (Choi et al., 2018; Lamela & Figueiredo, 2013; Milner et al., 2010; Yang et al., 2018), it was unexpected that in our study the association between internalizing problems and perpetrating abuse was not significant. However, in previous studies, internalizing problems have mostly been studied in isolation, without controlling for externalizing symptoms, even though internalizing symptoms and disorders (i.e., depression) often co-occur with externalizing symptoms/disorders (i.e., irritability, conduct disorder) (Angold et al., 1999; Cosgrove et al., 2011). Given the high correlation between internalizing and externalizing problems, the positive association between internalizing problems and perpetrating abuse in previous research (and also in the bivariate correlations in our study) might be inflated due to the overlap with externalizing symptoms. Another possibility is that the addition of internalizing problems to the model might have increased the association between externalizing problems and perpetrating abuse (suppressor effect, see Smith et al., 1992). However, since externalizing problems are correlated with perpetrated abuse independent of internalizing problems (see the bivariate associations in Table 3), it seems unlikely that a suppressor effect would be responsible for the significant association between externalizing problems and perpetrated abuse in the structural equation model. A systematic review of internalizing and externalizing problems recommends that research on internalizing or externalizing problems should measure both dimensions and that they should be controlled for each other in the analyses to address their overlap (Achenbach et al., 2016). By including both internalizing and externalizing problems in one model, we were thus able to disentangle which of the factors mainly drives the association with transmission of abuse, namely externalizing problems.

We did not find explicit or implicit emotion regulation to be associated with experienced or perpetrated child abuse. This is in contrast to meta-analytic evidence, which suggests that experienced childhood abuse is associated with emotion dysregulation (Lavi et al., 2019). Interestingly, Lavi et al. (2019) showed that the link between experienced childhood abuse and emotion dysregulation was moderated by study design, with stronger effects in cross-sectional studies compared to longitudinal ones. The authors speculate that the association with emotion regulation decreases over time, possibly due to regulatory skills, which are gained with age. In our study emotion regulation was assessed in adulthood, well after the time of experienced abuse. Participants in our sample might have acquired more emotion regulation techniques over time, which in turn could explain why it was not significantly associated with experienced childhood abuse.

The lack of association between emotion dysregulation and (the risk of) perpetrating child abuse is also not in line with our expectations, which were based on previous findings (Crouch et al., 2018; Miragoli et al., 2020; Rodriguez et al., 2017; Smith et al., 2014; Teisl & Cicchetti, 2008). The absence of a significant association between emotion regulation and experienced/perpetrating child abuse might be explained by how we operationalized emotion regulation. Lavi and colleagues' recent meta-analysis (2021) included studies on general dysregulation, dysfunctional coping and problem-solving, and impulsivity. Our study, however, focused on three dysfunctional coping strategies (i.e., rumination, catastrophizing, and self-blame), as well as the level of distraction during a goal-oriented task. Other aspects of emotion regulation, such as impulsivity, might be more strongly associated with perpetrating abuse (Lavi et al., 2021). Lastly, in our study, we have assessed general emotion regulation (strategies), rather than parenting-related emotion regulation, which has been suggested to modulate the link between parenting-related emotions and parenting behavior (Leerkes & Augustine, 2019). Future research may investigate whether parenting-related ER explains the link between having experienced and perpetrating child abuse.

### Strengths and Limitations

Our study is the first to investigate the mediating role of internalizing and externalizing symptoms in the association between childhood abuse and perpetrating child abuse in the same model. Furthermore, we studied implicit and explicit emotion regulation to clarify whether the operationalization of emotion regulation relates differently to experienced and perpetrated child abuse. Lastly, the multi-informant assessment method of measuring childhood abuse reduces the influence of reporter bias.

Nevertheless, this study does not come without limitations. First, reported associations are correlational, meaning we cannot infer causality in this cross-sectional design. Future research could investigate causal mechanisms using Mendelian randomization (Warrier et al., 2021) or examine the directionality of effects using longitudinal models. Second, since the sample largely consisted of Caucasian individuals, with an average age of 51 years (range 26–88 years), the results may not generalize to other populations (e.g., from other ethnicities). Thirdly, as the average levels of experienced abuse were not very high in our sample (average 1.5 on a scale of 1–5), this possibly limits the generalizability to more high-risk samples. However, there was still substantial variation in average levels of abuse, especially with regard to the experienced physical aggression scores, with values ranging up to the highest level (5 out of 5). The low average scores are largely due to the combination of multiple items from two scales and from different reporters, not precluding the presence of high abuse scores on separate items or individual reports.

### Clinical Implications

Elucidating mechanisms in the intergenerational transmission of childhood abuse can inform us about prevention and treatment strategies. As we found evidence that externalizing rather than internalizing symptoms partly mediate the association between experienced and perpetrated abuse, prevention and treatment strategies to reduce childhood abuse could focus on reducing externalizing problems in children or parents with a history of childhood abuse. Furthermore, externalizing problems could also function as a signal to therapists to pay extra attention to the parenting practices of clients who have experienced childhood abuse. However, since our findings are based on cross-sectional data, we cannot claim that externalizing symptoms developed after experiencing childhood abuse or preceded the perpetration of child abuse. Longitudinal studies with a prospective design including parents and their children are needed to establish a timeline and disentangle the potential role of externalizing problems in the intergenerational transmission of abuse. Future research could investigate how to prevent individuals with experiences of childhood abuse from developing externalizing symptoms, as well as which protective factors might prevent parents with externalizing symptoms from perpetrating child abuse. For example, social support may reduce the risk for individuals with a history of childhood abuse and current externalizing problems to abuse their own children. Targeted prevention and treatment programs for parents having experienced abuse in their childhood, inspired by this insight, may reduce the risk of the intergenerational transmission of abuse.

### Conclusion

Taken together, externalizing (but not internalizing) problems partially mediate the link between having experienced and perpetrating child abuse, for both mothers and fathers. Therefore, targeting externalizing problems may be a useful approach in prevention and treatment strategies to break the intergenerational transmission of abuse.

## Supplemental Material

Supplemental Material - The Role of Psychopathology and Emotion Regulation in the Intergenerational Transmission of Childhood Abuse: A Family Study Supplemental Material for The Role of Psychopathology and Emotion Regulation in the Intergenerational Transmission of Childhood Abuse: A Family Study by Cosima Nimphy, Marie-Louise Kullberg, Katharina Pittner, Renate Buisman, Lisa van den Berg, Lenneke Alink, Marian Bakermans-Kranenburg, Bernet Elzinga, and Marieke Tollenaar in Child Maltreatment
