# Supplementary material for: The Role of Psychopathology and Emotion Regulation in the Intergenerational Transmission of Childhood Abuse: A Family Study
Source: Child Maltreat. 2024 Feb 1;30(1):82–94. doi: 10.1177/10775595231223657 (PMC11656633; doi:10.1177/10775595231223657)
Supplement: Supplemental Material - The Role of Psychopathology and Emotion Regulation in the Intergenerational Transmission of Childhood Abuse: A Family Study [file sj-pdf-1-cmx-10.1177_10775595231223657.pdf]

Supplementary Material

Figure S1. Model with four mediators in the link between experienced and perpetrated abuse

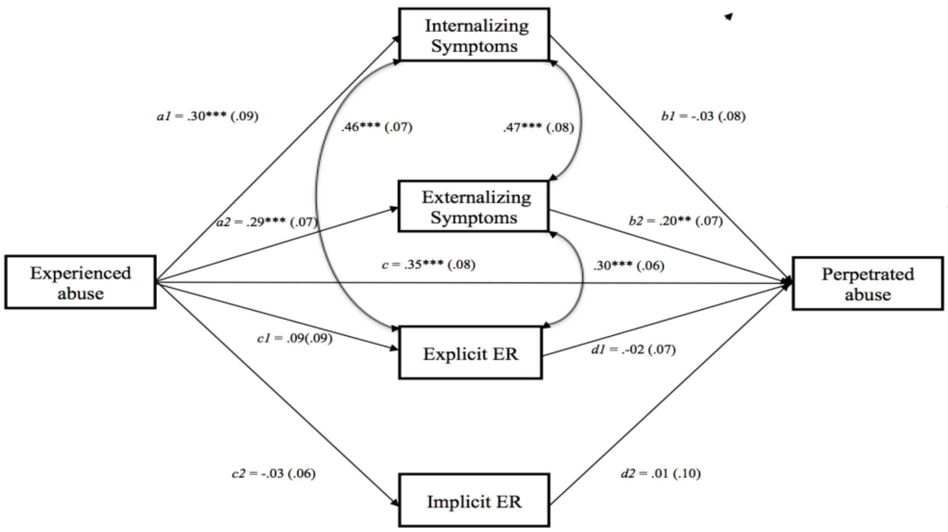

Notes. Adjusted standard errors in parentheses, * = $p < .05$, ** = $p < .01$, *** = $p < .001$, indirect effects: ab1 = - .01 (.03), ab2 = .06 (.03)*, cd1 = - .002 (.01), cd2 = .000 (.004), total effect = .40 (.07)***. Model is controlled for sex, age, and SES. *CFI* = 0.934. *RMSEA* =.065.

S1 Table. Occurrence of self-reported experienced emotional and physical abuse.[a, b]

	Never	Once	More than once
Abuse	9 (4%)	15 (6%)	226 (90%)
Physical Abuse	34 (14%)	43 (17%)	173 (69%)
Emotional Abuse	18 (7%)	26 (10%)	206 (82%)

[a]Children reported about father and mother.
[b]Occurrences are based on items describing parenting behaviors rather than the overall scales.
